# Current Status and Future Perspectives of Mass Spectrometry Imaging

**DOI:** 10.3390/ijms140611277

**Published:** 2013-05-28

**Authors:** Surendra Nimesh, Susantha Mohottalage, Renaud Vincent, Prem Kumarathasan

**Affiliations:** 1 Inhalation Toxicology Laboratory, Environmental Health Science and Research Bureau, Healthy Environments and Consumer Safety Branch, Health Canada, Ottawa, ON K1A0K9, Canada; E-Mails: surendra.nimesh@hc-sc.gc.ca (S.N.); renaud.vincent@hc-sc.gc.ca (R.V.); 2 Analytical Biochemistry and Proteomics Laboratory, Environmental Health Science and Research Bureau, Healthy Environments and Consumer Safety Branch, Health Canada, Ottawa, ON K1A0K9, Canada; E-Mail: susantha.mohottalage@hc-sc.gc.ca

**Keywords:** MALDI imaging, proteins, lipids, metabolites, mass spectrometry, tissues, matrix, toxicology

## Abstract

Mass spectrometry imaging is employed for mapping proteins, lipids and metabolites in biological tissues in a morphological context. Although initially developed as a tool for biomarker discovery by imaging the distribution of protein/peptide in tissue sections, the high sensitivity and molecular specificity of this technique have enabled its application to biomolecules, other than proteins, even in cells, latent finger prints and whole organisms. Relatively simple, with no requirement for labelling, homogenization, extraction or reconstitution, the technique has found a variety of applications in molecular biology, pathology, pharmacology and toxicology. By discriminating the spatial distribution of biomolecules in serial sections of tissues, biomarkers of lesions and the biological responses to stressors or diseases can be better understood in the context of structure and function. In this review, we have discussed the advances in the different aspects of mass spectrometry imaging processes, application towards different disciplines and relevance to the field of toxicology.

## 1. Introduction

Mass spectrometry imaging (MSI) is emerging as a powerful tool to scan biomolecular profiles in tissues, cell cultures and even on fingerprints. In tissue imaging, homogenization and separation steps are avoided to retain the spatial distribution of analytes within the tissue. Mass spectrometry imaging is an alternative approach to immunohistochemistry (IHC) and fluorescent microscopy. Immunological methods are limited to molecules for which antibodies are available and can suffer from experimental artifacts, due to the cross-reactivity of the antibodies. Fluorescence microscopy and IHC-based analyses require prior knowledge of the target analytes. Furthermore, simultaneous or multiplex analysis of multiple targets can be challenging. By permitting simultaneous detection of multiple analytes in a sample, MSI has a clear potential for high-throughput and high-content screening of analytes, including peptides, proteins, lipids and small metabolites, such as drugs and endogenous metabolites. Amongst all the available techniques, matrix assisted laser desorption ionization-time of flight (MALDI-TOF) has been widely used in performing MSI analysis. Caprioli *et al.*, in 1997, were the first to propose an application of MALDI-MSI to proteomics [1]. Owing to its potential, MSI has been successfully employed in the identification of protein, lipid biomarkers in cancer research, neuroscience, reproductive biology and pharmaceutics for the study and development of new drugs [2–5]. This review focusses on the advances in the methodology of mass spectrometry imaging and its applications in various areas of research with the potential use in toxicology.

## 2. Methodological Advances in Sample Preparation

**Figure 1:**
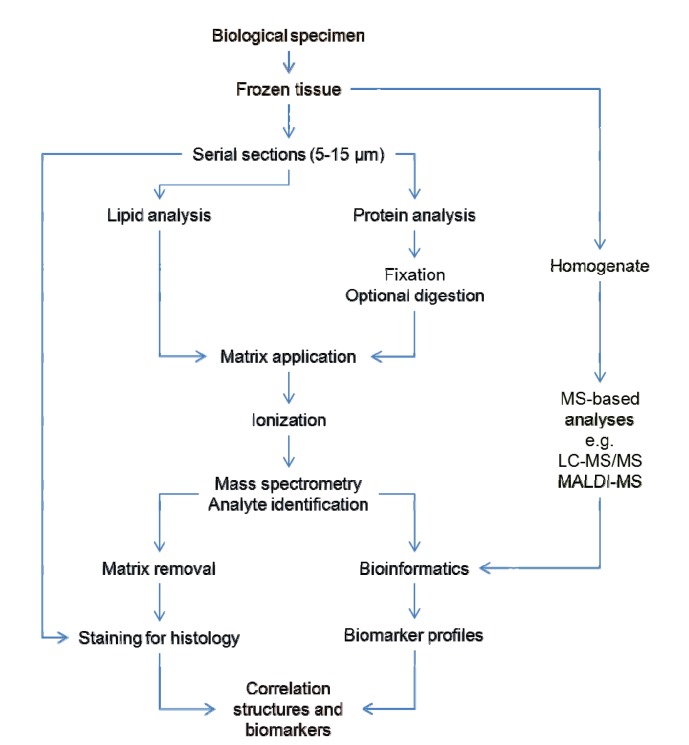
Workflow associated with matrix assisted laser desorption ionization-mass spectrometry imaging (MALDI-MSI) analysis, alongside complementary MS-based strategies and post-processing of data for biomarker analysis.

Animal organs, embryos, plant tissues, single cells and bacterial colonies have been analysed by MSI [6–12]. A variety of sample preparation strategies are compatible with this technique, which has been applied to fresh, alcohol preserved, snap-frozen, formaldehyde-fixed and paraffin-embedded tissues from brain, kidney, lungs, heart, various types of tumor samples, human biopsies and tissues from surgery [13–15]. For the preservation of sample morphology and spatial distribution of analytes and to minimize degradation, immediate freezing upon sample acquisition is preferred. However, freezing can lead to sample cracking and fragmentation, as different parts of the tissue will freeze at different rates, and can result in the formation of ice crystals. Moreover, use of specimen cassettes or plastic storage tubes for freezing samples can further distort the overall shape of the tissues. To avoid deformation and degradation of samples, snap freezing is highly desirable where the tissue may be loosely wrapped in aluminum foil and slowly plunged into freezing liquid, such as nitrogen, ethanol, isopropanol or isopentane, and kept at −40 °C or −80 °C, depending on the type of freezing liquid. This freezing procedure preserves the morphology of the tissue and also prevents degradation of the biological content [16]. Prolonged storage at −80 °C can affect the integrity of the frozen tissue samples. The workflow for sample preparation for MSI analysis includes tissue sectioning, loading on the support target plates, fixation to the support, optional on-tissue proteolytic digestion, application of the matrix and mass spectrometry analysis (Figure 1).

### 2.1. Tissue Sectioning

For routine analysis of samples by MSI, frozen tissue sections are usually cut on a cryostat at 5–15 μm thickness [16]. Tissue thickness is of major concern. Although thick sections are easier to manipulate, they need a longer duration to dry and may not be electrically conductive after loading onto a MALDI slide, resulting in a poor quality spectra [16], while it is difficult to manipulate very thin sections and tearing can occur easily. Thin tissue sections cut at 5–15 μm have been shown to be preferable for analysis of large molecular weight molecules in the range of 3–20 kDa [17]. Frozen tissue blocks are mounted on the cryostat head employing embedding medium, such as agar or optimal cutting temperature polymer (OCT). However, care should be taken to avoid contamination of the tissue sections with OCT, because its components can lead to ion suppression during mass spectrometry analysis by MALDI-TOF-MS. The disposable blades used for sectioning are often packaged with a very thin film of a lubricant, which has been shown to be a potential source of sample contamination leading to poor data quality. Therefore, the blades are rinsed with methanol and acetone prior to tissue sectioning [16].

### 2.2. Tissue Loading on Target Plates

Tissue samples can be mounted on aluminum, stainless steel and gold-coated MALDI target plates [6,18,19]. Gold-coated plates offer high contrast to the dull surface of the tissue along with clear identification of tissue margins, as well as comparably better binding of the tissue section. However, application of such opaque plates limits the microscopic visualization of tissue sections. Recently, novel conductive glass slides and MALDI-friendly staining protocols have been developed [20]. These glass slides are usually coated with a very thin (~130 Å) film of indium-tin oxide, rendering them electrically conductive with uniform maintenance of high voltage potential in the ion source of MALDI-TOF mass spectrometers. Tissue sections mounted on conductive glass slides can be stained for regular histopathological visualization prior to analysis by MSI [20]. Several nuclear dyes, including cresyl violet, offer good staining qualities without interference in the MSI analysis. There are also reports on histological stains of two consecutive sections where any stain can be used. Hematoxylin and eosin (H & E) stain is commonly used for this process. The main disadvantage with this process is that the MALDI image is obtained from a section different from the one that is stained. The third approach involves initial MALDI imaging followed by matrix removal and H & E staining [20]. This is comparably a more favourable approach.

### 2.3. Fixation

Fixation of the tissue to the support plate followed by rinsing prior to the deposition of the matrix significantly enhances the quality of the acquired images [20–23]. The usual fixation steps consist of a brief 70% ethanol wash followed by a 90%–100% ethanol wash to dehydrate and temporarily fix the tissue [16,20,21]. After fixation, the total ion yields have been reported to increase by a factor of 3–10-fold, depending on the type of tissue investigated. For protein analysis, lipids and salts are removed from the tissue surface by washing in a solution containing alcohol and water. For protein analyses in tissues with high lipid content, delipidation by washing with organic solvents, such as chloroform or xylene, without delocalization of analytes is recommended [17,24]. For lipid analyses, washing with solvents are not recommended; however, there are several published rinsing procedures prior to lipid MSI analyses that have shown improvements [25–28]. For instance, Wang *et al*. employed 150 mM ammonium acetate solution for washing the tissue, so as to get rid of the salts and other contaminants, including OCT compound, prior to lipid profiling [25]. Another study reported the use of cold 50 mM ammonium formate, pH 6.4, or ammonium acetate, pH 6.7, for washing mouse brain tissue sections, followed by negative ion mode MALDI-MSI for lipid profiling, exhibiting significant enhancement in signal intensity and the number of identified analytes [27]. Thomas *et al.* have reported on serial washes of mice fetus tissue sections by conventional organic washes, followed by aqueous-based buffer washes and have demonstrated enhanced improvement in sensitivity and selectivity on the detected proteins [29]. Different types of biomolecules will require different treatments, and the initial fixation and washing procedure need to be adapted and optimized for the specific MSI application. After rinsing, the sample plates are dried prior to optional on-tissue digestion and/or matrix application.

### 2.4. On-Tissue Digestion for Proteomic Analysis

High molecular weight proteins are often not detected in MALDI experiments, due to their low abundance, poor ionization and low detection efficiency [30]. It is also possible that detection of higher molecular weight proteins by MSI can be adversely affected by the ability to solubilize them from the tissues. On-tissue proteolytic digestion can be performed to bring these large proteins into the detectable mass regions [17,31]. This is achieved by application of a proteolytic enzyme, such as trypsin, onto the surface of the tissue sections. For optimum enzyme activity, the tissue sample has to be wet and incubated at 37 °C for a time period from one hour to overnight, depending on the analyte. Excess liquid on the tissue surface can lead to diffusion of analytes during incubation. To minimize fluid volume and to prevent diffusion of peptides, the enzyme can be applied by spray coating or direct spotting, keeping in mind that the size and distribution of enzyme spots will limit the spatial resolution of the MSI image [17,31]. Protein digestion generates small peptides in the range of 400–3500 Da, a range where most instrumental sensitivity and resolution are high and can improve protein identification by subsequent MS/MS analyses [30].

### 2.5. Matrix Application

#### 2.5.1. Types of Matrices

Mounting and fixation of the tissue sample on the support plate is followed by the application of matrix for mass spectrometry analysis. It is highly imperative to choose the right matrix and optimize analysis parameters, in order to obtain high quality mass spectral data from tissue samples along with spatial information of the analytes. The most commonly used matrices include 3,5-dimethoxy-4-hydroxycinnamic acid (sinapinic acid, SA), α-cyano-4-hydroxycinnamic acid (CHCA) and 2,5-dihydroxybenzoic acid (DHB). Sinapinic acid is commonly used for high molecular weight proteins, while CHCA is preferred for low molecular weight peptides. In a comparative study, comprised of SA, CHCA and DHB, SA yielded the best combination of crystal coverage and signal quality [16]. Washing the tissue section prior to matrix application, as described above, significantly improves the quality of spectra obtained with SA as the matrix. Furthermore, SA at matrix concentrations >30 mg/mL compared to 10 or 20 mg/mL solution yielded high quality spectra [16]. A solvent composition consisting of 50:50 acetonitrile/water or ethanol/water with 0.3%–1% TFA yielded consistently good results on a wide variety of tissue samples [16].

Lipid analytes have been observed to exhibit uncontrolled fragmentation, resulting in a loss of specificity and sensitivity. For instance, gangliosides, which are comprised of a ceramide backbone with attached sialylated oligosaccharides, when exposed to MALDI, easily loses the sialic acid residues [32,33]. Hence, matrices used for lipid MSI have to be different from those employed for proteins. A mixture comprised of matrix, dihydroxyacetophenone (DHA), heptafluorobutyric acid (HFBA) and ammonium sulfate, was shown to remarkably suppress lipid cationization, while yielding high resolution imaging of sphingomyelin (SM) and phosphatidylcholine (PC) species [34]. Further, 9-aminoacridine (9-AA) was shown to be a suitable matrix for *in situ* analysis of phospholipids and sulfatides in rat brain tissue sections [35]. There are solvent-free matrix deposition methods used in MSI analysis of lipids [36]. Recently, matrices have been proposed for imaging lipids by mass spectrometry containing a combination of DHB with aniline, pyridine or 3-acetylpyridine, allowing analyses in both positive and negative ionization [37]. Also, Dong *et al.* have reported on enhanced improvement in the analyses of phospholipids by MSI using 1,5-diaminonaphthalene as the matrix [38].

In terms of MSI analysis of drugs, their metabolites and endogenous metabolites, CHCA, DHB, DHA or 9-AA are commonly used [39,40]. Shanta *et al.* have reported on a new combination of matrix using 3-hydroxycoumarin and 6-aza-2-thiothymine for small molecules analyses [41]. Nanoparticles derived from metals, such as Au, Ag, Pt, Zn and Ti, have also been employed as matrix, which facilitates MALDI-MS detection of small molecules and metabolites [42,43]. Gold nanoparticles coated onto mouse brain tissue allowed detection of several metabolites, including neurotransmitters, fatty acids, nucleobases and glycosphingolipids (GSLs), such as minor molecular species of sulfatides and gangliosides [44,45]. Also, TiO_2_ nanoparticles used in nanoparticle-assisted laser desorption/ionization imaging MS (Nano-PALDI-IMS) efficiently detected endogenous low molecular weight metabolites in mouse brain (80–500 Da) that were hardly detectable using DHB matrix [46]. The use of nanoparticle-derived matrices in MSI applications is evolving.

#### 2.5.2. Matrix Deposition

Several different strategies have evolved for deposition of matrix onto tissue surface. Spotting is one such method, where matrix is delivered as small droplets onto the tissue, while coating refers to the delivery of a homogeneous layer of matrix over the entire tissue. In both approaches, multiple matrix applications are required to cover the entire tissue section. However, excess loading of matrix can suppress analyte signals [47]. Furthermore, to check the uniformity of the matrix loading and to rectify uneven matrix depositions, an internal calibrant consisting of a peptide in the same mass range as the analytes of interest can be added to the matrix solution [48]. Although the best results have been obtained when the samples were analyzed promptly, matrix-spotted tissue sections can be stored overnight in a desiccator with minimal loss of signal.

For spotting, two commonly available automated devices with different types of droplet ejectors are available, which are inkjet-style piezo nozzles and focused acoustic dispensers. These ejectors are capable of dispensing 100 pL droplets for a spot size 100–150 μm diameter on the tissue upon drying. Since spotted matrix is generally larger than the focused laser spot, the surface area wetted by the droplet dictates the maximum attainable spatial resolution [49], meaning that the resolution is dependent on the spacing of the printing. One of the advantages of using automated spotting devices is that they allow multiple rounds of matrix deposition, at the same precise location to increase analyte extraction from the tissue. Matrix spotting devices, such as chemical inkjet printers (ChIP from Shimadzu, Japan), are equipped with inkjet-style piezo nozzles for delivery of droplets 55 μm in diameter and 87 pL in volume [50]. Another device employed for automated matrix deposition is a desktop inkjet printer with a six-channel piezoelectric head that dispenses 3 pL droplets [51]. Spotting devices, such as the Acoustic Reagent Multispotter (Labcyte Inc., Sunnyvale, CA, USA), utilize acoustic energy to facilitate matrix deposition [21].

The strategy for matrix deposition by spray coating of matrix solution onto the tissue surface typically uses pneumatic and electrospray deposition. Comparison of various matrix deposition strategies, such as electrospray, airbrush and inkjet, revealed that the mass spectral images obtained from inkjet-printed tissue specimens were of better quality and more reproducible than from specimens prepared by the electrospray and airbrush methods [51]. However, inkjet dispensers have the disadvantage of clogging the capillary, when spotting highly concentrated matrix solutions. Matrix can be coated by using ImagePrep (Bruker Daltonics), a device that generates a fine mist of matrix droplets using vibrational vaporization of the matrix with a piezoelectric spray head, where concentration can be varied and controlled. The ImagePrep produces fine droplets of 25 μm diameter, resulting in a homogeneous matrix layer, which further leads to high lateral resolution and good mass spectra [52]. Another spray system routinely employed for matrix deposition is the TM sprayer™ (Leap Technology) [53]. The spray nozzle of the TM sprayer™ creates a fine solvent mist that is carried by a heated inert gas and instantaneously evaporates upon contact with the MALDI plates. This instrument provides a large selection of spray pattern designs for MALDI-MSI.

Another strategy consists of vapor-phase deposition of matrix via sublimation, which results in uniform coating of matrix over the sample plate and has been shown to be useful for imaging of low molecular weight molecules, particularly, phospholipids [36]. The instrumentation for sublimation is comprised of sublimation glassware, heated bath and vacuum pump. The matrix deposition using this technique is highly reproducible and can easily be controlled with time, temperature and pressure settings. Improved image profiling with this strategy is attributed to uniform deposition of highly purified matrix in microcrystalline morphology. DHB, CHCA and 1,5-dianimonapthalene (DAN) matrices have been generally deposited using the sublimation technique [36,54].

### 2.6. Mass Spectrometry and Data Analysis

Advances in mass spectrometric instrumentation have empowered high-throughput peptide and protein identification. A number of techniques have been developed to drive the mass spectrometers to perform MSI, with MALDI, desorption electrospray ionisation (DESI) or laser ablation electrospray ionisation (LAESI) as the means of generating ions [55,56]. The UltrafleXtreme™ (Bruker Daltonics) MALDI-TOF-TOF-MS platform is an example of a useful configuration for MSI [29]. The TOF-MS was introduced by Stephens *et al*. in 1946, and since then, it has been coupled with MALDI and secondary ion mass spectrometry (SIMS) ion sources [57]. Some of the advantages of TOF-MS include high sensitivity (low femtomole to attomole levels for proteins and peptides), good transmission ratio (50%–100%), wide dynamic mass range and fast repetition rate [57]. The utility of MALDI-TOF for MSI was first demonstrated by Caprioli *et al*. in the mapping of insulin in a tissue section of rat pancreas [1]. Other mass analyser configurations used in MALDI-MSI include ion-trap/orbitrap and FT-ICR configurations [39,58]. These high resolution mass analysers are useful in terms of achieving MS images with high mass resolution and accuracy, which will be advantageous in terms of protein identification. High resolution MALDI-MSI was also employed to map distribution of neuropeptides within different cell clusters of rat, mouse and human pituitary tissue sections [59]. The stigmatic imaging mass spectrometer employed produced ion-count images with pixel sizes of 500 nm with a resolving power of 4 μm, which resulted in higher image contrast compared to those from microprobe imaging experiments [59]. Furthermore, these technologies can lead to rapid targeted biomarker identification, namely, by enabling multiplex MSI methodologies [60].

During mass spectrometric analysis, a multitude of measurements are done in a pre-defined sequence, in a selected tissue section. The mass spectrum is acquired at each discrete spatial spot, a pixel to create an ion image of various analyte peaks in the range of several hundred or few thousands of *m*/*z* values. Typical MSI data can be considered as a collection of several such spectra across the tissue. Computational analysis of these *m*/*z* data is therefore needed, and multiple sequential methods are used for analysis of these data. The major steps involved in analysis include (1) preprocessing: baseline removal, spectra normalization and noise reduction [61–63]; (2) data reduction using mass spectrometry peak picking or scale-space transformations [63,64]; (3) data presentation: employing multivariate statistics, e.g., principal component analysis (PCA) [65,66], spatial segmentation of a MALDI-imaging data set on the basis of spectra clustering [61,67], supervised classification of spectra of a MALDI-imaging data set [68,69] and for determination of discriminative *m*/*z* values; (4) post-processing, e.g., image magnification and co-registration with a high-resolution microscopy image.

Preprocessing of MALDI-imaging data allows cleaning of the spectra from the baseline and noise, followed by selection of peaks encoding relevant information. The initial step of data preprocessing includes spectra normalization, baseline correction and realignment or recalibration of spectra. Normalization of ion intensities is done to minimize spectrum-to-spectrum differences in peak intensities that could be due to sample variability, sample preparation, instrument variation and experimental error. The process of normalization compiles all data onto a common intensity scale to facilitate direct comparisons of spectra. Total ion count (TIC) is one of the most commonly employed normalization method in MSI, where all mass spectra are divided by their TIC, so as to obtain a dataset with a similar area under the spectrum. Deininger *et al.* proposed comparison of the images after TIC and median normalization for proper selection [62]. Baseline correction is a usual method of mass spectra preprocessing and can be performed by employing the top-hat operator from mathematical morphology. The baseline correction function is integrated into the software supplied by the instrument manufacturers, *i.e.*, Data Explorer, Flex Analysis or BioMap. The advanced baseline correction function in Data Explorer employs peak width, flexibility and degree, which can be optimized. However, it generally allows good estimation of the baseline for only a narrow *m*/*z* range of about 10 kDa. To improve upon this limitation, processing of each spectrum is done multiple times using correction parameters optimized for different *m/z* regions followed by combination into a final spectrum [63]. Flex Analysis offers a convex hull option that works well across the full *m*/*z* range without any user input, but this package imports only native data files and cannot process data from other instruments. Biomap provides baseline models using values from the first and second derivatives of each spectrum, but with certain threshold criteria. Baseline correction is a standard method of mass spectra preprocessing. Spectra normalization and baseline correction are followed by a reduction of the data set by selecting peaks appearing in at least 1% of spectra. The next crucial step is an edge preserving denoising of *m*/*z* images for each *m*/*z* value from the selected peaks. Data acquisition during MALDI-imaging also generates noise that can be identified by visual inspection of *m*/*z* images corresponding to selected *m*/*z* values. Denoising has been performed in order to reduce this pixel-to-pixel variability, adjusting the level of denoising to the local noise level and to the local scale of the features to be resolved [61]. The smoothing process assists in peak detection, as well. Smoothing can be done with different types of filters, such as the Gaussian or Savitzky Golay algorithm. The Flex Analysis employed the Savitzky Golay algorithm for smoothing of peaks.

Spectral realignment employs a subset of peaks common to most sample spectra, using the criteria that a peak must be found in more than 90% of the spectra [63]. It is desirable to identify common peaks that span the whole mass range of interest. Further, these common features are used as arbitrary calibrants for spectrum realignment using a quadratic calibration algorithm. Matlab provides an alignment algorithm, msalign, which shifts the spectrum and computes a cross correlation of the spectrum with a theoretical spectrum generated using user provided alignment criteria; another study reported use of alignment of peaks with respect to the mean spectrum [70,71].

**Figure 2:**
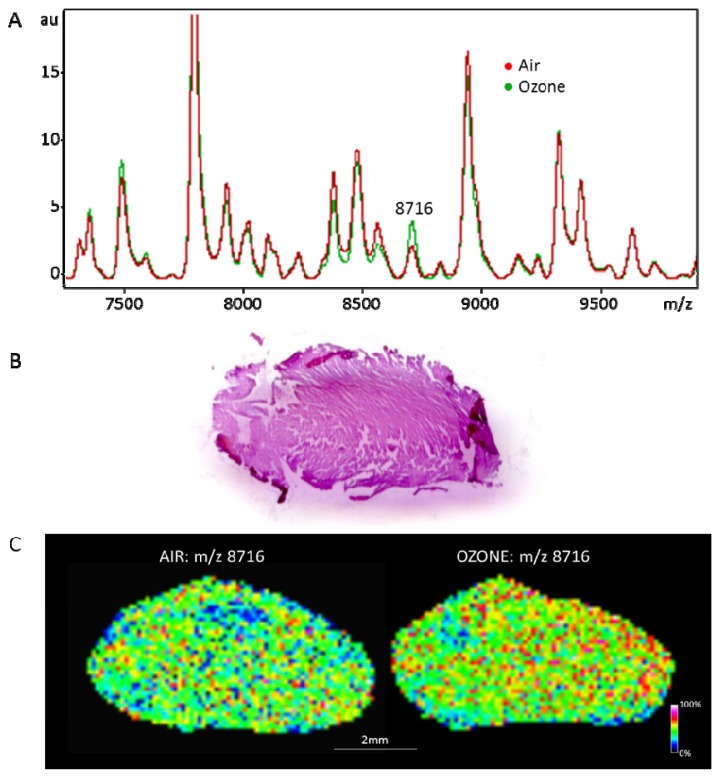
Normalized (to the total ion count) average MALDI-TOF mass spectra (**A**), histology data (**B**) and normalized mass spectral images (UltrafleXtreme, Bruker™) of heart sections (**C**) from ApoE-/- mice exposed to air (controls) and 0.8 ppm ozone (treatment). Frozen heart sections (12 μm thickness) on indium tin oxide (ITO) coated slides were prepared in triplicate for mice from control and treatment groups (*n* = 4/group) for MSI analysis. Sinapinic acid was used as the matrix and was spray coated on the dried tissue section (Bruker ImagePrep) for imaging. Detection was in a linear positive mode. Lateral resolution for imaging was 100 μm. One of the analyte peaks among those that were significantly different (*p* < 0.05) with ozone treatment compared to the control group was tentatively assigned as small-inducible cytokine B10 (*m*/*z* 8716).

The next step consists of data presentation employing multivariate statistical analysis of reduced and processed spectra, and the results are displayed as a spatial segmentation map. Typically, mining of the high resolution *m*/*z* data is conducted using various bioinformatics software tools. For example, the FlexImaging 2.0 software (Bruker Daltonics) allows color-coded visualization for the spatial distribution of ions detected during MSI (Figure 2). Software packages, such as ClinPro Tools (Bruker Daltonics), allow performance of multivariate statistics, including principal component analyses (PCA), spatial segmentation of image data based on hierarchical clustering or variance ranking that allows class imaging [67,72]. Recently, a bio-statistical approach, termed as principal component analysis-symbolic discriminant analysis (PCA-SDA), was developed to identify biomarkers and successfully applied to prostate cancer tissue samples [73]. In addition, MS/MS analysis of parent ion from imaging analysis, especially with the on-tissue digestion step and matching of fragment ion data against public domain databases (e.g., NCBInr, SwissProt), can lead to analyte identification.

A recent strategy involves generation of molecular networks from mass spectrometry data obtained by nanospray desorption electrospray ionization (nanoDESI) mass spectrometry for direct chemical monitoring of living microbes [74]. The molecular networks allows visualization of observed molecules as familial groupings in which similarities within the mass spectrometry fragmentation data are assessed via vector correlations and displayed as an MS/MS network. This integrated approach comprising of two different methodologies provides a powerful workflow for direct chemical analysis of secreted microbial exchange factors in live colonies. For instance, mass spectral molecular networking of *Pseudomonas* sp. strain SH-C52 in conjunction with the peptidogenomic strategy allowed the detection and partial characterization of thanamycin, a chlorinated non-ribosomal peptide synthetase-derived peptide with antifungal activity [74,75].

Another study employed Ingenuity Pathway Analysis (IPA) for functional analysis of the identified metastasis-specific proteins, from patients with papillary thyroid carcinoma (PTC) [76]. MSI in combination with 1D-gel electrophoresis and mass spectrometry, followed by IHC validation, lead to identification of *m/z* species that specifically distinguished metastatic from non-metastatic tumors, among which *m/z* 11,608, 11,184 and 10,094 were identified as thioredoxin, S100-A10 and S100-A6, respectively. Interestingly, IPA discovered a strong relationship of all candidate proteins with the TGF-β-dependent epithelial-mesenchymal transition (EMT) pathway [76].

## 3. Applications of MSI

Owing to its relative simplicity, MSI has been employed in several investigations, such as analysis of distribution of biomolecules in biological compartments, as well as in different regions of tissues in healthy and disease conditions.

### 3.1. Systemic Distribution of Biomolecules

Most of the early MSI studies were aimed at the identification of proteins in tissue sections. These studies were critical in demonstrating the overall correlation between protein distribution and different stages of growth, stress or pathological conditions. Although MSI is capable of simultaneously measuring *m*/*z* ratios of several hundred proteins, most of the early reports focussed on the superimposition of the mass spectral pattern onto the histology data.

There are two distinct strategies for MSI analyses of biomolecule distribution. The bottom-up strategy consists of the breakdown of large proteins into smaller fragments by digestion with proteolytic enzymes prior to MSI, while the top-down strategy enables detection of intact proteins directly from tissue sections without enzymatic digestion [31,77]. In addition, one of the major drawbacks of MALDI-MS is mass-dependent sensitivity and resolution drop-off. Notwithstanding, a distinct advantage of the top-down approach is that it can directly determine the abundance of protein forms, as intact proteins are less susceptible to experimental artifacts of sample processing and can provide valuable information on post-translational modifications (PTM). The top-down approach has been successfully employed in targeted studies for detection of single proteins, usually <100 kDa [4].

Caprioli *et al*. analyzed tissue sections by blotting on specially prepared targets containing C-18-coated resin beads [1]. In another study, mouse colon tissue sections were blotted on a conductive polyethylene membrane [78]. Direct MSI analysis yielded over 100 peptide/protein signals in the mass range of 2–30 kDa, with 30–50 having relatively high signal intensities [78].

Lipids are the building blocks of cell membranes and are involved in signaling pathways [79]. The utility of liquid ionic matrices (LIMs) to map lipids within tissue sections was shown by deposition of DHB/3-acetylpridine on a whole rat brain tissue section and imaging in both positive and negative ion modes [37]. Lipids have been imaged using various MS techniques, such as MALDI, desorption electrospray ionization (DESI) and secondary ion mass spectrometry (SIMS) [80,81]. Owing to the presence of polar head groups, the lipids can be easily ionized [82]. Lipids, such as PC, SM and cholesterol, are ionized in positive ion mode, whereas phosphatidylinositols, phosphatidylserines and sulfatides are ionized in negative ion mode [83]. However, phosphatidylethanolamine (PE) can be detected in both positive, as well as negative ion modes [34,84,85].

In another study, MALDI imaging was used for visualization and molecular imaging of latent fingerprints (LFPs) [86]. Gold nanoparticles (AuNPs) generated from argon ion sputtering aggregated on the ridges and grooves of LFPs in two different forms, exhibiting two contrasting colors (*i.e*., pink on ridges and blue on grooves), arising due to different surface plasmon resonance (SPR), allowing clear visualization of LFPs on different substrates, including plastic, glass and paper. Laser irradiation of a gold nanoparticle-coated LFP resulted in generation of ions at *m*/*z* 227, 241, 253, 255, 281 and 283, which were assigned to various lipids [86]. The laser desorption/ionization property of the gold nanoparticles allowed direct analysis of endogenous and exogenous compounds embedded in latent fingermarks and imaging of their spatial distributions without affecting the fingerprint patterns [86].

In a recent study, Yagnik *et al.* explored a multiplex MSI methodology utilizing a linear ion trap-orbitrap hybrid mass spectrometer for imaging of LFPs fingerprints [39]. This strategy involves multiple smaller steps in each MALDI raster step, where the ions produced from the first step are being analyzed by the high mass resolution orbitrap mass analyzer; spectra from subsequent steps are acquired by the linear ion trap and are analysed either in MS or MS/MS mode. The chemical distribution of several known endogenous lipids in fingerprint residues were detected and imaged, including cholesterol (as a water loss at *m*/*z* 369.352), oleic acid (*m*/*z* 283.263) and various triacylglycerols (TAGs) (e.g., [TAG(45:1)+Na]^+^ at *m*/*z* 785.666 and [TAG(48:1)+Na]^+^ at *m*/*z* 827.713) [39]. Furthermore, some exogenous compounds were also detected, which includes benzyl dimethyl dodecyl ammonium (BDDA) at *m*/*z* 304.300, dimethyl dioctadecyl ammonium (DDA) at *m*/*z* 550.626 and verapamil at *m*/*z* 455.291 [39].

### 3.2. Pathologies

One of the major applications of MSI is determination of molecular changes that occur with disease progression. Usually, the study involves comparative proteomic analyses, whereby mass spectra (*m*/*z* peaks) of different tissue sections are correlated to disease information, such as known molecular events involved through histology analysis and the overall symptoms. Alzheimer’s disease, Parkinson’s disease, kidney diseases, muscular dystrophy, cardiovascular disease and cancer (lung, breast, ovarian, prostate, colon, liver) have been investigated by MSI [2,3,69].

In a recent study, MALDI-MSI was employed for multimodal imaging of acylcarnitines, PC, lysophosphatidylcholine (LPC) and SM from different microenvironments of breast tumor xenograft models [2]. The analyses revealed spatially heterogeneous distribution of lipids within the tumors. Viable tumor regions showed abundance of four lipid species, namely PC(16:0/16:0), PC(16:0/18:1), PC(18:1/18:1) and PC(18:0/18:1), whereas in necrotic tumor, only LPC(16:0/0:0) was detected. Further, a heterogeneous distribution of palmitoylcarnitine, stearoylcarnitine, PC(18:0/22:1) and SM(18:1/16:0) sodium adducts mostly co-localized with hypoxic tumor regions [2]. In another study, MALDI-MSI proteomic algorithms have been employed for the identification of human epidermal growth factor receptor 2 (HER2) status in breast cancer tissues [69]. Protein profiling by direct MSI analyses of breast cancer tissues that were predefined for HER2 status by IHC and fluorescence *in situ* hybridization was in good agreement with the HER2 overexpression. A clear distinction between HER2-positive and HER2-negative tissues was achieved on the basis of MSI profiling with high sensitivity values of 83%, specificity of 92% and all around accuracy of 89% [69]. Similarly, MSI analysis of human breast carcinoma tissue sections revealed three proteins specific to different regions of the cancer tissue, namely, thymosin β-4 in stroma, histone H2A in the ductal carcinoma *in situ* region and calgizzarin in invasive carcinoma [87].

In an attempt to identify new biomarkers for ovarian cancer, MALDI-MSI was used for imaging 20 tissue sections from 19 ovarian tumors, 10 being benign, 6 being carcinomatous and three being borderline [3]. During analysis of tissue profiles for peptides, carcinoma-specific peaks were observed at *m*/*z* of 3300 and 4800, while in the case of benign tissue, the analytes were observed in the *m*/*z* range from 1200 to 2000, with CHCA as a matrix. Small protein analysis employing SA as matrix revealed two specific signatures, one at *m*/*z* from 9500 to 14,000 and the other at 17,500. For high mass proteins, carcinoma-specific signatures were observed between *m*/*z* 23,000 to 25,000, while in the case of benign and borderline profiles, they were observed between *m*/*z* 65,000 and 68,000 [3]. Profiling of stage III and stage IV carcinoma by MSI followed by principal component analysis detected the established ovarian cancer protein biomarkers, 17.7 kDa tetranectin, 26.8 kDa kallikrein 5 precursor and 36.9 kDa urokinase plasminogen activator [3]. Similarly, other ovarian cancer studies using MSI include the work of Lemaire *et al.* and Kang *et al.* [4,88].

Image analysis of 22 prostate sections (11 with and 11 without prostate cancer) showed highly distinct protein expression between normal and tumorous regions of prostate tissue sections [89]. Another study with MSI allowed identification of a protein fragment of mitogen-activated kinase kinase kinase 2 (MEKK2) in prostate cancer tissues [90].

In a comprehensive study, MSI was employed to image tissue sections from 42 lung tumors, 34 being primary tumors, two pulmonary metastases of previously resected non-small-cell lung cancer (NSCLC), one pulmonary carcinoid, five metastases to the lung from other sites and eight normal lung samples [91]. Mass spectral profiles, consisting of more than 1600 distinct protein peaks, revealed differentially expressed proteins that enabled the discrimination of primary lung tumors from other sites and classify nodal involvement. However, from this large number of peaks, 15 distinct protein peaks allowed differentiation of patients with resected NSCLC with poor prognosis (median survival, six months, *n* = 25) and those with good prognosis (median survival, 33 months, *n* = 41, *p* < 0.0001) [91].

### 3.3. Drug Metabolism

Spatial localization of drugs and their metabolites is required for studies related to absorption, distribution, metabolism and excretion (ADME) in pharmacokinetics studies. One of the preferred techniques in whole animals is whole body autoradiography, which provides spatial resolutions of 10 μm. Mass spectrometry imaging provides detailed spatial information about the parent drug compound and the resulting metabolites in a single experiment, without the requirement for labelled analytes. MALDI-MSI was used for the first time in 1999 for the detection of the anti-tumor drug paclitaxel in rat liver sections and ovarian cancer biopsies. Later, the technique was extended to whole-body imaging of several drugs, including vinblastine, olanzapine, β-peptide, raclopride and terfenadine [19,92,93].

In one of the earlier studies on drug localization in tissue sections, the distribution of the antipsychotic drug, clozapine, was mapped in rat brain after dosing [5]. MSI analysis revealed the presence of the drug in the cerebral cortex and striatum, but not in the corpus callosum. A recent study employed MALDI-MSI for monitoring the distribution of two anti-cancer drugs, erlotinib and gefitinib (EGFR tyrosine kinase activity inhibitors) in three different lung tumors, planocellular lung carcinoma, adenocarcinoma and large cell lung carcinoma [94]. The drugs were found in a higher amount within the regions of stroma cells in contrast to the tumor tissue areas. Consistent correlations between the localization of compound signals and the planocellular tumors were observed, as the planocellular tumors are characterized by larger growth in local areas [94].

To elucidate the efficacy of the tuberculosis drug, moxifloxacin, distribution of the drug at different time points after administration to tuberculosis-infected rabbit was analysed by MALDI-MSI [95]. Accumulation of the drug was observed in granulomatous lesions at higher levels than that in the surrounding lung tissue, 1.5 h post-dosing. The highest levels of the drug were maintained between 1.5 and 3.25 h post-dosing. Data were validated by quantitative LC/MS/MS analysis of lung and granuloma extracted from adjacent biopsies from the same animals. Heterogeneous drug distribution was seen, with high levels observed within the granulomas and very low levels of drug observed in the caseum [95]. In another study, MALDI-MSI was employed to track and quantify the distribution of an inhaled reference compound, tiotropium, within the lungs of dosed rats [96]. Imaging profiles within 15 minutes after exposure showed dispersion of tiotropium ions (*m*/*z* 392.1) and fragment ions (*m*/*z* 170.1 and 152.1) in a concentration gradient (80 fmol to 5 pmol) in the lung parenchyma and pleura. The amount of the drug quantified by MSI was validated after chemical extraction of the drug from the lung tissues. The intensities of the obtained MSI images were in accordance with the histological images for the drug [96].

Imaging of whole-body sections of mice administered raclopride, a dopamine D2 receptor-selective antagonist, showed high intensities for drug ions at *m*/*z* 347, which later degraded to fragment ions detected at *m*/*z* 129 [97]. The spatial localization of the drug indicated the presence of raclopride in the liver, lung, brain and kidney, ten minutes after administration, which correlated well with autoradiography data.

### 3.4. Reproductive Biology

MSI has been used to study the growth, maturation and functioning of reproductive organs. Spermatozoa produced by the testes undergo maturation while in movement across the epididymis, which involves interaction with the epididymal fluid. The first study on mouse epididymis involved direct tissue analysis; caput and cauda were separated by blotting and contacting each section to the polyethylene membrane for 5 min. MSI revealed significantly different protein profiles in the caput and cauda regions of the epididymis [98]. Higher sensitivity was obtained with MSI conducted on fresh frozen tissues in contrast to tissues imprinted on blotting paper. MSI of whole epididymis tissue sections, laser capture micro-dissected cells and secretory products showed over 400 different proteins [99]. More than 50 proteins were observed to be spatially localized from caput to cauda within the epididymis. Furthermore, semi-quantitative information for each protein was obtained in correlation to the signal intensities observed in the different protein profiles.

A study done on mouse testes evaluated the spatial localization of different variants of seminolipids, which are sulfated glyceroglycolipids present in mammalian testes [100]. Seminolipids are synthesized in primary spermatocytes, and their expression remains stable during spermatogenesis. The MSI analysis demonstrated that various seminolipids were present during maturation of testes: a peak at *m*/*z* 795 (C16:0-alkyl-C16:0-acyl), present throughout the tubules, and a peak at *m*/*z* 767 (C16:0-alkyl-C14:0-acyl) were observed at the edge of tubules, where spermatogonia and spermatocytes are present, and another at *m*/*z* 809 (C17:0-alkyl-C16:0-acyl) was present in the inner lumen of tubules and was specifically expressed in spermatids and spermatozoa. This study suggests that the expression pattern of each molecular species of seminolipids is different during testicular maturation [100].

MALDI-MSI has also been applied for mapping the spatial and temporal distribution of PE species associated with mouse embryo implantation [101]. The negative ion MSI images of implantation sites revealed significant differences in the distribution of PE during days four to eight of pregnancy. At the end of day eight, MALDI-MSI images showed localization of docosahexaenoate-containing PE lipids (confirmed by negative ion CID) to apoptosis-destined regions (AM pole), while oleate- and arachidonate-containing PE lipids were localized to angiogenic regions (M pole). During embryo implantation, the processing of arachidonic acid generates cPLA2 and COX-2, which further leads to prostaglandin signaling, which mediate embryo attachment and uterine decidualization. During embryo implantation, COX-2 expression localizes to the M pole above the ectoplacental cone (EPC), and the MALDI-MSI of arachidonate-containing PE lipids exhibited strong correlation with cPLA2α and COX-2 [101]. In addition, Laggarigue *et al.* have also discussed the potential of MSI in the field of reproductive research [102].

### 3.5. Toxicology

Exposure to an environmental stress, such as a toxic chemical or radiation, can lead to enhancement of a group of protective or toxicity mechanisms orchestrated through upregulation or downregulation of gene or protein expressions [103,104]. Accordingly, the search for biomarkers associated with pollutant exposures has been attempted using various approaches, including mass spectrometry, LC-fluorescence, LC-coulometric array detectors and other biochemical analysis methods [105–109].

One of the initial studies employed desorption electrospray ionization (DESI) MS for imaging LFPs from individuals exposed to small amounts (5 μg) of drugs of abuse, such as cocaine and D9-tetrahydrocannabinol (D9-THC, psychoactive component from cannabis), and explosives, such as trinitrohexahydro-1,3,5-triazine (RDX, high-energy explosive) [110]. DESI images of LFPs allowed identification of chemicals transferred to glass, paper or plastic surfaces along with retention of spatial resolution of 150 μm. Some of the components identified in LFPs by DESI imaging include the distribution of cocaine (*m*/*z* 304) on glass, fatty acid cis-hexadec-6-enoic acid (*m*/*z* 253) on glass, ^37^Cl-RDX (*m*/*z* 257) on plastic and Δ9-THC (*m/z* 313) on paper [110]. MALDI-MSI has been used to image the endogenous distribution of lipids from fresh and aged, groomed and un-groomed fingermarks [111]. Fingermark patterns were reconstructed by retrieving the *m*/*z* values of oleic acid and its fragments, which allowed a distinction between the three aged fingermarks. MSI allowed clear distinction between the fresh and aged fingermarks, as well as different storage conditions, where oleic acid (*m*/*z* 283.27) degradation was observed with the increase in temperatures between 48 °C, 37 °C and 60 °C [111]. Further, to emphasize MALDI-MSI being a non-destructive technique, a simple washing protocol (submerging the support in 70:30 ACN/0.1% TFA solution) was adopted, which exhibited a fingermark that could be further investigated with classical forensic approaches [111].

Bradshaw *et al.* explored MALDI-MSI for simultaneous detection of both endogenous and exogenous substances to the analysis of fingermarks contaminated with condom lubricants, so as to empower forensic scientists to provide further support to the evidence in alleged cases of sexual assault [112]. The MALDI-MSI analysis of condom contaminated fingermarks enabled the detection and spatial mapping of the same ion series, as detected in the condom extract, and was assigned to the presence of sodiated and potassiated polyethylene glycol (PEG). Additionally, endogenous molecules, such as the fatty acids, oleic acid (*m*/*z* 283.2) and cholesterol (*m*/*z* 369.2), were also identified [112]. Further, the usefulness of this technique was extended by analysing condom-contaminated fingermarks after several weeks, which exhibited images with very clear ridge details. Further, for the improvement and imaging of latent fingermarks, a two-step matrix application method, named as the “dry-wet” method, was developed, where the matrix was dusted onto the sample, followed by solvent spray using a robotic device [113]. The dry-wet method was reported to be reproducible and superior, compared to conventional spray-coating deposition. Also, MALDI-MSI was employed for detection of endogenous peptides and small proteins in groomed fingermarks from a cohort of 80 donors (40 females and 40 males), by rubbing the fingers on the forehead, nose and chin, and the MS data were analysed by the Partial Least Squares Discriminant Analysis (PLS-DA) method that enabled sex determination with 85% accuracy [114]. Such analysis can be useful in addressing specific questions in forensic toxicology, such as identification of the sex of the person associated with the crime.

MALDI-MSI analyses of latent fingerprints have been successfully implicated in identification of various endogenous and exogenous compounds. As described previously, in the case of individuals exposed to RDX and cocaine, MS imaging enabled identification of both the exogenous materials, as well as the endogenous distribution of fatty acids and TAGs. Hence, MSI can constitute a strategy for instant analysis of biochemical changes that occur in the fingermarks of individuals exposed to radiation or chemical contaminant exposure.

In a recent study, MALDI-MSI was employed to search for induced protein changes in various brain regions of adult rats following neonatal exposure to the cyanobacterial toxin, β-N-methylamino-L-alanine (BMAA) [115]. The MALDI-MSI analyses revealed that animals with neuronal lesions and astrogliosis within the CA1 region had the most severely affected hippocampus. Decreases in the level of proteins involved in energy metabolism, such as cytochrome c oxidase and its subunit, cytochrome c oxidase polypeptide VIIa, was observed in the CA1 and the DG regions of hippocampus at the lowest dose (150 mg/kg) of BMAA, without histopathological lesions being detected in these brain regions. Exposure to a higher dose (460 mg/kg) also induced changes in the expression of S100β, histones, calcium and calmodulin-binding proteins and guanine nucleotide-binding proteins [115].

Our preliminary results on MSI analysis of hearts from atherosclerosis-prone (ApoE-/-) mice exposed to air and 0.8 ppm ozone exhibited significant protein expression changes in ozone exposed animals compared to their corresponding air controls (Figure 2). These findings indicate that MSI analysis is capable of identifying pollutant exposure-related protein changes amongst the changes relevant to developing atherosclerosis pathology in these mice. Although such investigations can be valuable in identification of health risks associated with various pollutant exposures, the area of toxicological research using MSI is still at its infancy and needs to be advanced through future work in this field. A combination of protein, lipid and metabolite analysis by MSI in cells and tissues can permit the understanding of the mode of action of a contamination through a systems biology approach and aid in better risk analysis in toxicology.

## 4. Conclusions and Future Perspectives

Mass spectrometry imaging is rapidly emerging as an indispensable tool for molecular imaging of biological samples from cells, tissues sections or whole animal body sections. In several studies, MSI has allowed identification of new molecules as signatures of diseases or the effect of drugs and toxicants. The technique empowers the investigation and spatial localization of both identified and unidentified molecules without any need for labeling or contrasting agents, which further facilitates discovery of new biomarkers and their validation. We have discussed some of the potential applications of MSI, with emphasis on the methodology and advances in the sample preparation procedures and explored the applicability of MALDI-MSI in investigation of molecular events caused by environmental toxicant exposure. Although MSI analysis requires a sophisticated and costly analytical platform, this approach is relatively simple and, in combination with classical histological methods, can provide new insights into simultaneously occurring biological processes that could not be obtained otherwise. MSI can easily be tailored for high-throughput analysis, while maintaining the spatial resolution of the analytes of interest, which can be achieved with current advanced analytical platforms available for high-speed mass spectrometry analyses. Future sophistication of MSI instrumentation, improved sample processing methodologies and new bioinformatics tools will further the capability of this technique, and open up avenues to advance its application in the field of toxicology.
